# Supplementary material for: Maturation and substrate processing topography of the Plasmodium falciparum invasion/egress protease plasmepsin X
Source: Nat Commun. 2022 Aug 4;13:4537. doi: 10.1038/s41467-022-32271-7 (PMC9352755; doi:10.1038/s41467-022-32271-7)
Supplement: Supplementary file 1 — Supplementary Information [file 41467_2022_32271_MOESM1_ESM.pdf]

Supplementary information

Maturation and substrate processing topography of the *Plasmodium falciparum* invasion/egress protease plasmepsin X

pH	Alpha helix (%) Mean ± S.D.	Beta sheet (%) Mean ± S.D.
7.5	10.88 ± 0.58	29.62 ± 0.82
5.5	3.71 ± 0.83 *	30.88 ± 1.10

Supplementary table 1: Percentages of alpha helices and beta sheets in recombinant PM X as estimated from circular dichroism spectra at pH 7.5 and 5.5. Values were obtained by using the online server K2D3 (http://cbdm-01.zdv.uni-mainz.de/~andrade/k2d3/). Experiment was repeated twice. Shown are the mean values with standard deviations. Data were analyzed statistically by two-tailed Student's t-test *: $p = 0.014$. Source data are provided as a Source Data file.

Primer #	Sequence	Notes
151	CCGAAAAGTGCCACCTGACGTCTATACG AGTAAAACACATCACAGAT	Forward primer for PCR of AMA1 5' UTR
152	AGAGGGCTAATGCGTTTCATCTCGAGTTT TTGTACAATTTATAACAA	Reverse primer for PCR of AMA1 5' UTR
153	TTGTTATAAATTGTACAAAAACTCGAGATG AAACGCATTAGCCCTCT	Forward primer for PCR of PM X ORF
154	CAAGAGCAAAAAGTAAAAACCCTAGGCCA GGTGCAGCACATT	Reverse primer for PCR of PM X ORF
155	CCAGGTGCAGCACATTATGCAGCAATGA GTAAAGGAGAAGAAC	Forward primer for PCR of GFP ORF
156	TCGTCCTTGTGCGGCCGCTTATTTGTACA GTTCATCCATG	Reverse primer for PCR of GFP ORF
157	CAAATGCACAATTAGATGAAAAAAACTTT GCTGCATTTACTACGTATAATAAAAATAA	Mutagenesis primer for NFAA mutant in pEOE-PM X-GFP
158	CTGACCATTTATCTGATGCCGCAAAACAT GTGTATGAACAAG	Mutagenesis primer for SDAA mutant in pEOE-PM X-GFP
159	TTCAGATAATAGTTCGGCCGCAAAAAATT TTATTGCTGCTG	Mutagenesis primer for SSAA mutant in pEOE-PM X-GFP
160	GTTCGATTGAAAAAAATTTTATTGCTGCTG CAAATAAAAACGCTACAGTAGAGCAAA	Mutagenesis primer for IAAA mutant in pEOE-PM X-GFP
161	TTAAAGGGTTAAAATGCACCTTAGATTTTA CTACGTATAATAAAAAT	Mutagenesis primer for delA mutant in pEOE-PM X-GFP
162	CTTTTTAGATTTTACTACGGCTGCAGCTG CAGCTGCAAAAAATTTTATTGCTTT	Mutagenesis primer for delB mutant in pEOE-PM X-GFP
163	AAAGGGTTAAAATGCACCTTAGAAAATAA AAACGCTAC	Mutagenesis primer for delPD mutant in pEOE-PM X-GFP
164	AGCATCGAGAAAAACTTTATCGCCGCTGC AAACAAGAACGAAACCGTGGAACAGA	Mutagenesis primer for IAAA mutant in mammalian system
165	GAGAACAATTCTGACAACAGCAGCAGCG CTGCAAAAAACTTTATCGCCCTGGAAAAC	Mutagenesis primer for SSAA mutant in mammalian system
166	TGGACGAGAAGAACTTCGCCGCATTCAC CACCTACAACAAG	Mutagenesis primer for NFAA mutant in mammalian system
167	CAGTGTACCCCATCTTCGGAACCGGCAG CACCAATGT	To make catalytic dead PM X in mammalian system

Supplementary table 2: List of primers used in this study. All primers were purchased from Integrated DNA technologies.

Name of the antibody	Clone	Source	Catalog # (for commercial ab)	Lot # (for commercial ab)	Dilutions used	Application
Rat anti-FLAG	L5	Novus Biologicals	63730L	B227795	1:1000	Western blot
Rabbit anti-HA	polyclonal	Sigma	H6908	0000097652	1:1000<br>1:500	Western blot<br>IFA
Mouse anti-HA	16B12	Biolegend	901501	269834	1:1000<br>1:500	Western blot<br>IFA
Mouse anti-GFP	JL-8	Takara	632381	A8034133	1:1000<br>1:500	Western blot<br>IFA
Rabbit anti-AMA1	polyclonal	LSBio	LS-C369007	71053	1:500	Western blot<br>IFA
Rabbit anti-His	Polyclonal	Sigma	SAB4301134	492635533	1:1000	Western blot
Mouse anti-RAP1	2.29	The European Malaria Reagent Repository			1:500	IFA
Mouse anti-MSP1	12.4	The European Malaria Reagent Repository			1:500	IFA
Rabbit anti-EBA-175	MRA-2	MR-4			1:500	IFA
Mouse anti-PM V	23.1.2	Ref. 1			1:500<br>1:50	Western blot<br>IFA
Rabbit anti-SUB1	Polyclonal	Ref. 2			1:1000	Western blot
Rabbit anti-Sera5	24C6.1F1	Ref. 3			1:1000	Western blot
Mouse anti-RON4	24C6.4F1 2	Ref. 4			1:500	IFA
Rabbit anti-Rh5	polyclonal	Ref. 5			1:500	Western blot

Supplementary table 3: List of primary antibodies used in this study. References for non-commercial antibody source:

1. Banerjee, R. *et al*. Four Plasmepsins are active in the Plasmodium falciparum food vacuole, including a protease with an active-site histidine. *Proc. Natl. Acad. Sci. U. S. A. 99* (2), 990– 995 (2000).
2. Blackman, M.J. *et al*. A subtilisin-like protein in secretory organelles of Plasmodium falciparum merozoites. J Biol Chem. 273(36):23398-409 (1998).
3. Delplace, P., Dubremetz, J.F., Fortier, B., Vernes, A. A 50 kilodalton exoantigen specific to the merozoite release-reinvasion stage of Plasmodium falciparum. Mol Biochem Parasitol 17: 239–251 (1985).
4. Roger, N. *et al*. Characterization of a 225 kilodalton rhoptry protein of Plasmodium falciparum. Mol Biochem Parasitol 27(2-3):135-41 (1988).
5. Nagaoka, H. *et al.* Antibodies against a short region of PfRipr inhibit Plasmodium falciparum merozoite invasion and PfRipr interaction with Rh5 and SEMA7A. Sci Rep. 10(1):6573 (2020).

a

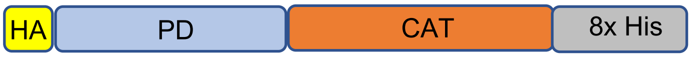

b

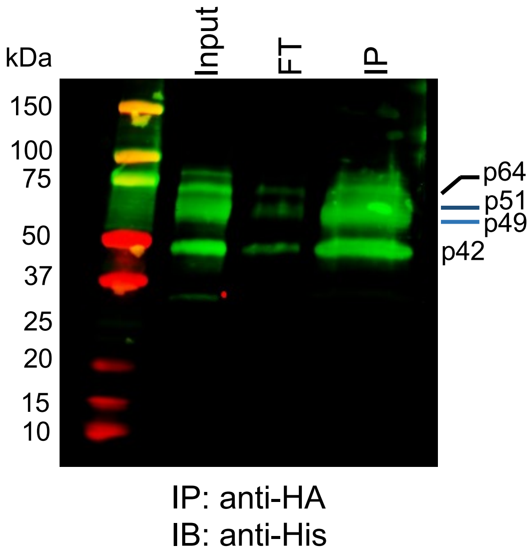

Supplementary Fig. 1. N-terminal part of the prodomain (PD) stays bound to the processed forms of PM X. a Schematic of the recombinantly expressed PM X (rPM X) in mammalian cells with a hemagglutinin (HA) tag at the N terminus and 8 histidine tag (8x His) at the C terminus. **b** Western blot with anti-His antibody. rPM X as depicted in (**a**) was immunoprecipitated with anti-HA antibody followed by western blot. FT: Flow through, IP: immunoprecipitated fraction. Indicated are the molecular weights for the polypeptide bands. PD: prodomain, CAT: catalytic domain. Experiment was repeated two times. Shown is a representative blot. Source data are provided as a Source Data file.

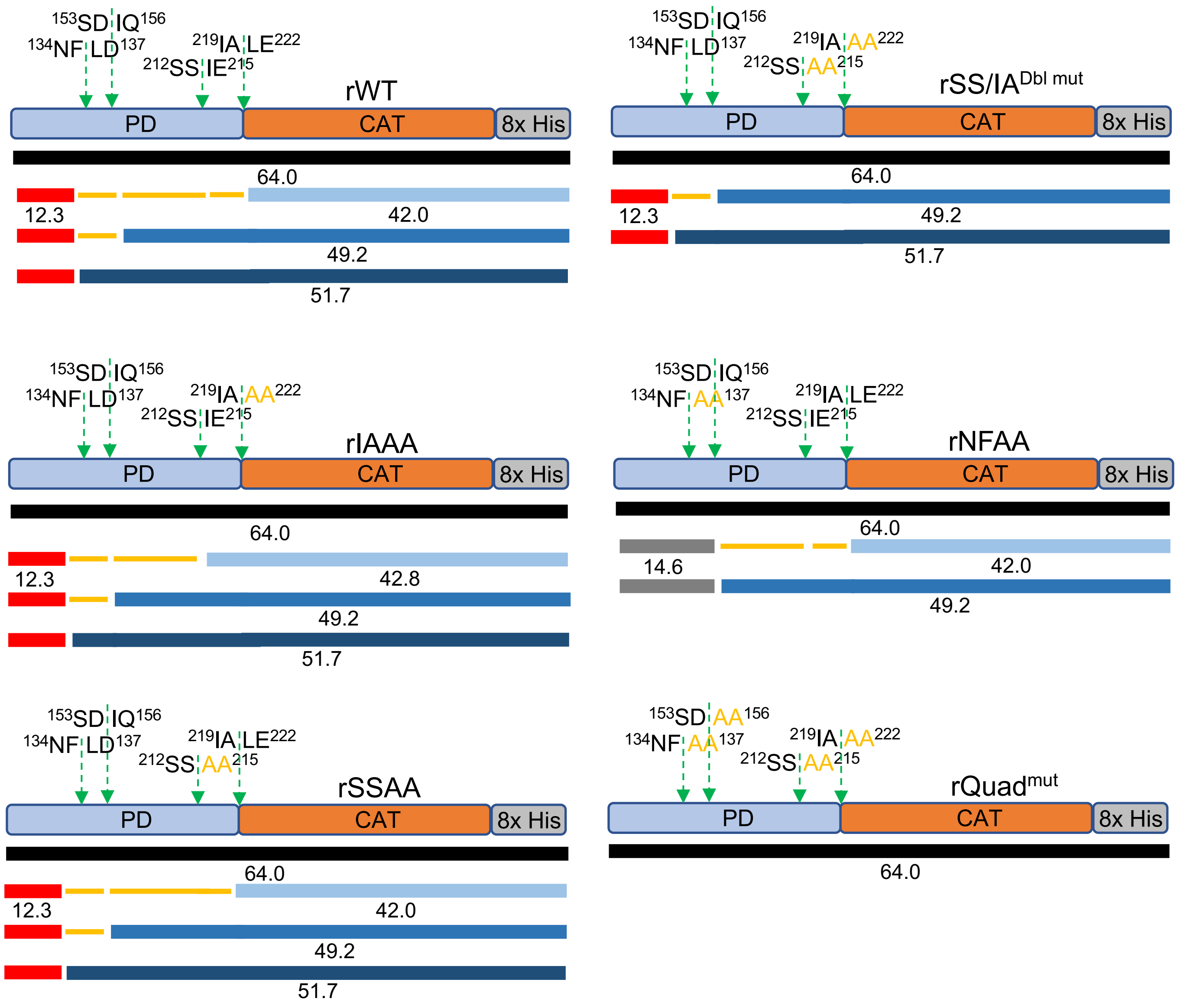

Supplementary Fig. 2. Schematics of the wild type (WT) and the mutant constructs used in this study for recombinantly expressed PM X (rPM X) in mammalian cells. Think lines Indicate the processed fragments that were detected either by western blot or by Coomassie blot. Numbers represent the calculated molecular weights in kDa. Orange thin lines represent processed bands not detected. Tetrapeptide sequence flanking the PM X autocleavage sites are indicated with positional information. For mutant constructs point mutations are represented by orange alphabets. Green dotted arrows represent the scissile bonds. PD: prodomain, CAT: catalytic domain.

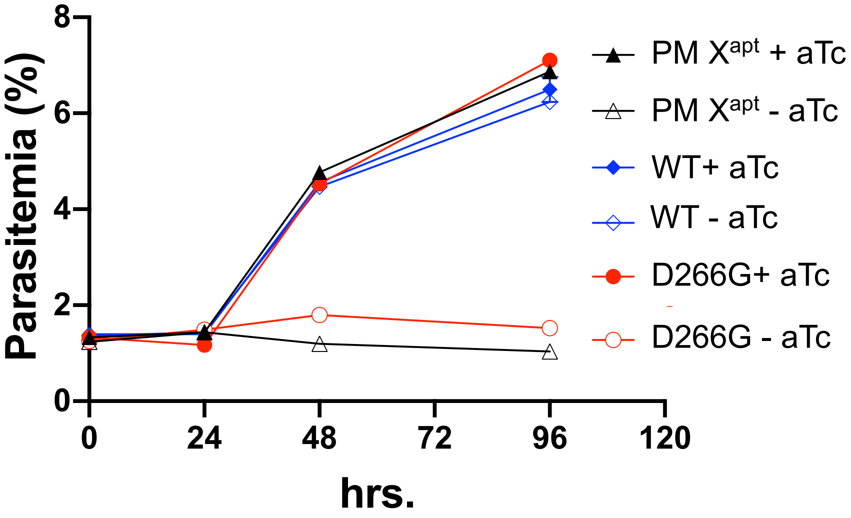

Supplementary Fig. 3. A wild type (WT) but not a catalytic dead (D266G) form of second copy PM X could rescue the growth defect due to knockdown of endogenous PM X. PM X^{apt} parasites complemented with a second copy of the PM X gene, wild type (WT) or catalytic mutant (D266G) were grown in presence or absence of aTc. Parasitemia were determined at indicated times. Mean values from three independent experiments are shown and error bars represent standard deviations. Source data are provided as a Source Data file.

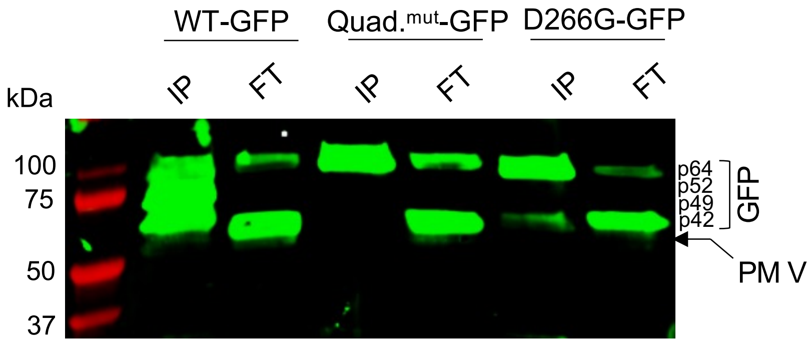

Supplementary Fig. 4. Pulldown of second copy PM X-GFP from parasites. WT, Quad^{mut} and D266G PM X-GFP were immunoprecipitated from equal numbers of 42-45 h schizonts using anti-GFP antibody. Samples were used for *in vitro* substrate cleavage assay presented in Fig 3. Post reaction, a fraction of the pulled down protein was analyzed by western blot with anti-GFP antibody. IP: immunoprecipitated fraction, FT: flow through. Plasmepsin V (PM V) was used as a marker for flow through. Experiment was repeated for three times. Shown is one representative blot. Source data are provided as a Source Data file.

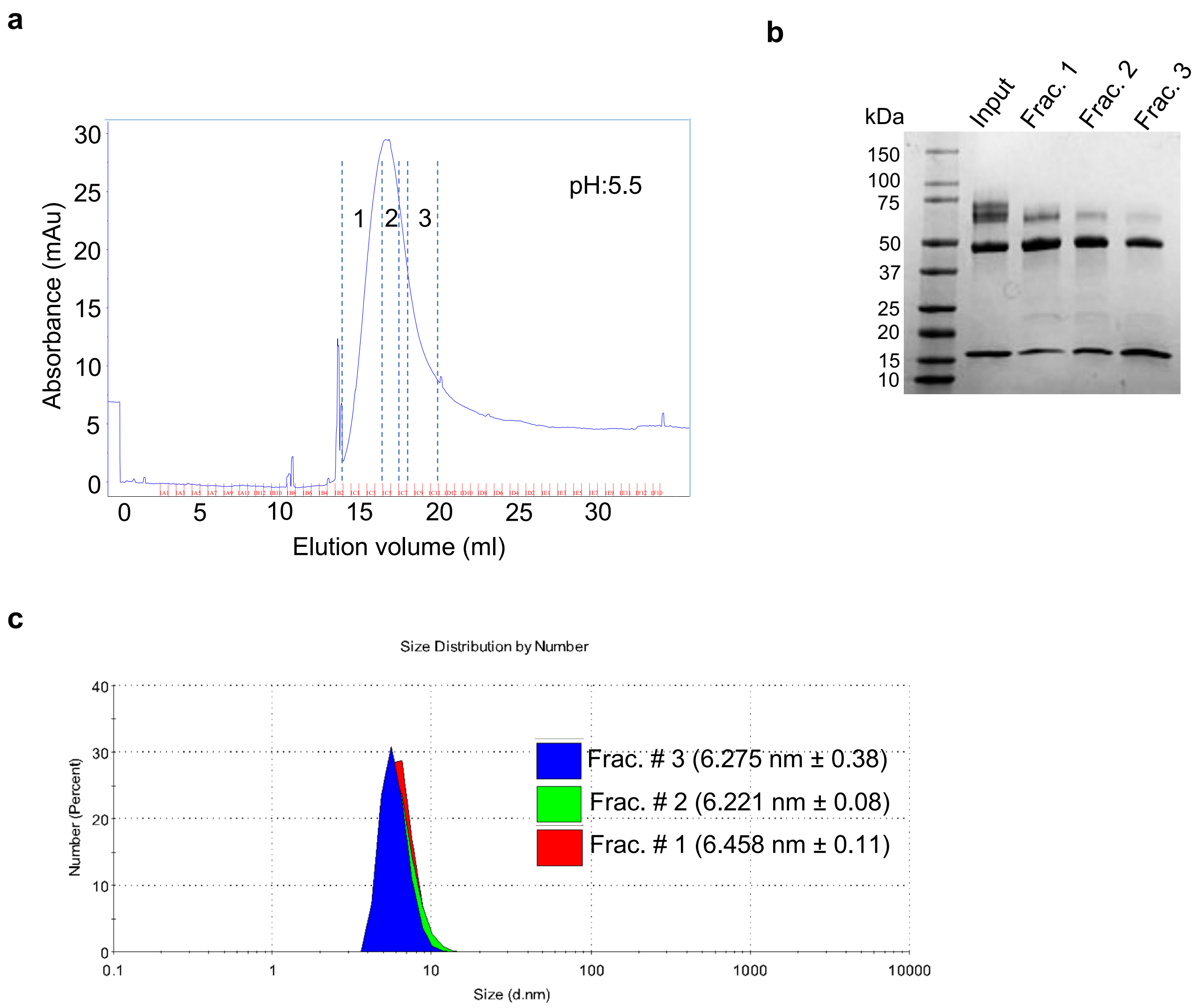

Supplementary Fig. 5. The N-terminal part of the prodomain (PD) remains attached with the rest of PM X at pH 5.5. a Elution profile of rWT PM X during size exclusion chromatography. Numbers within dotted lines indicate the fractions that were collected to run on SDS-PAGE followed by Coomassie staining **(b)** and dynamic light scattering (**c**). **c** The size distribution (diameter in nanometer unit) of PM X molecules of the three fractions collected in (**a**) and subjected to dynamic light scattering (DLS). Fractions are color coded. Numbers within parenthesis represent mean diameters from two separate experiments with standard deviations. Based on the crystal structure (PDB: 7RY7) the calculated mean diameters for rPM X with or without PD are 6.4 nm and 5.2 nm respectively. Details about the calculation are in the methods section. Both experiments (**b** and **c**) were performed two times. Source data are provided as a Source Data file.

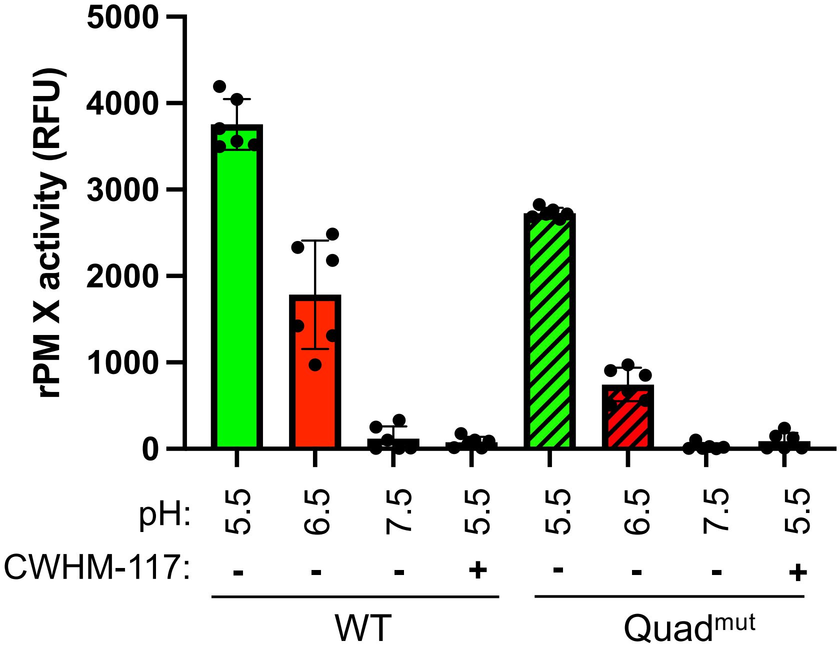

Supplementary Fig. 6. *In vitro* substrate cleavage activity of the wild type (WT) and quadruple mutant (Quad^{mut}) rPM X under different pH conditions. As substrate, a fluorogenic synthetic peptide that corresponds to the PM X cleavage site in Rh2N was used. 1 uM substrate peptide was incubated with 25 ng of rPM X at 37°C for 40 minutes. The increase in relative fluorescence intensity (RFU) over time due to cleavage by PM X was measured. To inhibit PM X activity, 1 uM CWHM-117 was added to samples at pH 5.5. Mean values from three independent experiments are shown and error bars represent standard deviations. Source data are provided as a Source Data file.

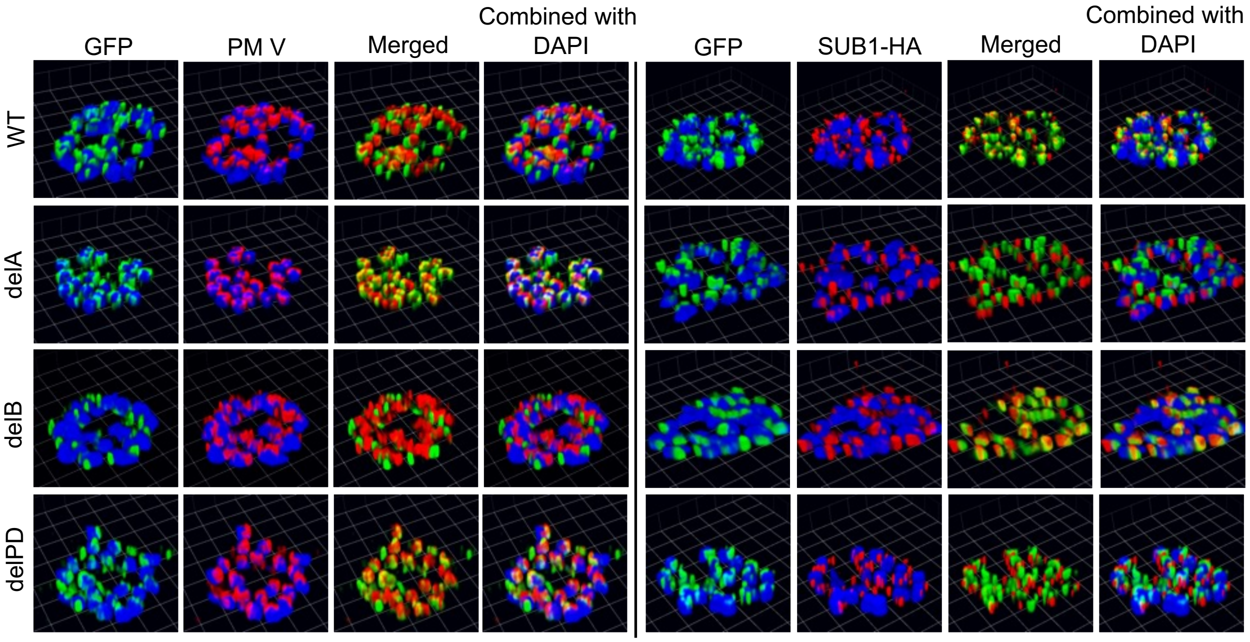

Supplementary Fig. 7. Different prodomain (PD) truncation mutant forms of PM X-GFP traffic differentially inside the parasites. 3-dimensional snapshots of the same images as in Figure 5b captured by confocal Airyscan microscopy. Each grid line is 1.61 µm.

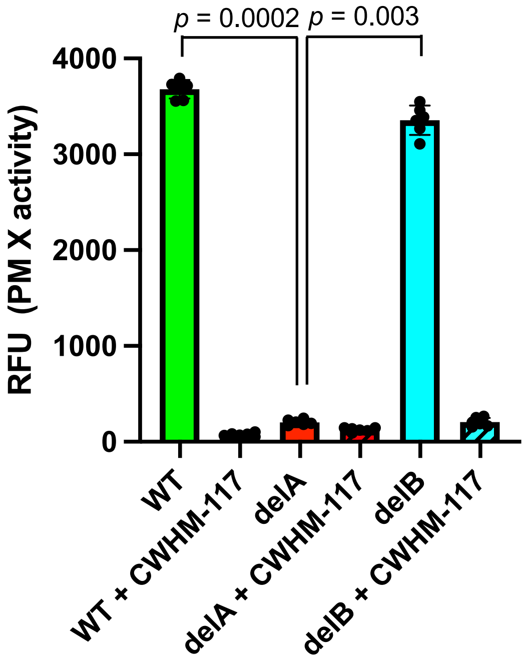

Supplementary Fig. 8. *In vitro* activity of prodomain (PD) mutant forms of parasite-derived PM X. Second copy PM X tagged with GFP was purified from schizonts using anti-GFP antibody. Isolated enzyme was then incubated with substrate as in fig. 3. Reaction was carried out for 1 h at 37°C. Mean values from two independent experiments are shown and error bars represent standard deviations. Data were analyzed statistically by two-tailed Student's t-test. ***: $p < 0.001$. Source data are provided as a Source Data file.

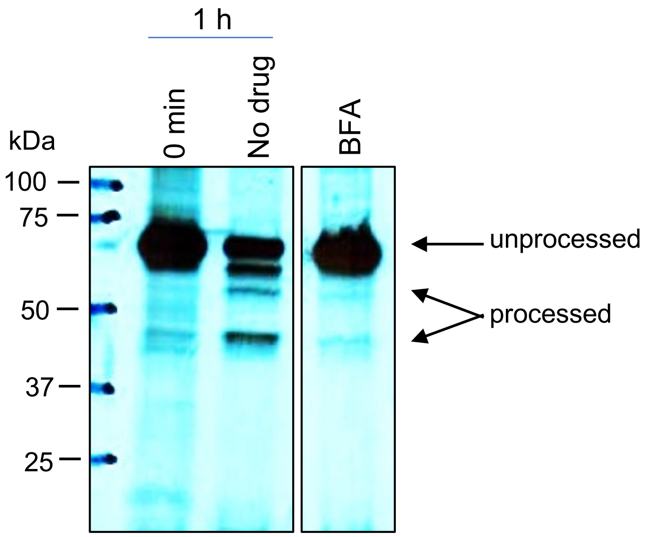

Supplementary Fig. 9. PM X processing is brefeldin A (BFA)-sensitive. 40-43 h schizonts expressing PM X-3xHA were pulse labelled with [^{35}S]methionine-cysteine for 5 min followed by chasing in unlabeled medium in the presence of cycloheximide (10 µg/ml). To one sample BFA (5 µg/ml) was added during chase. After 1 h, PM X was pulled down using anti-HA beads followed by autoradiography. The precursor and the processed forms of PM X are indicated. Experiment was performed two times, shown is one representative autoradiograph.

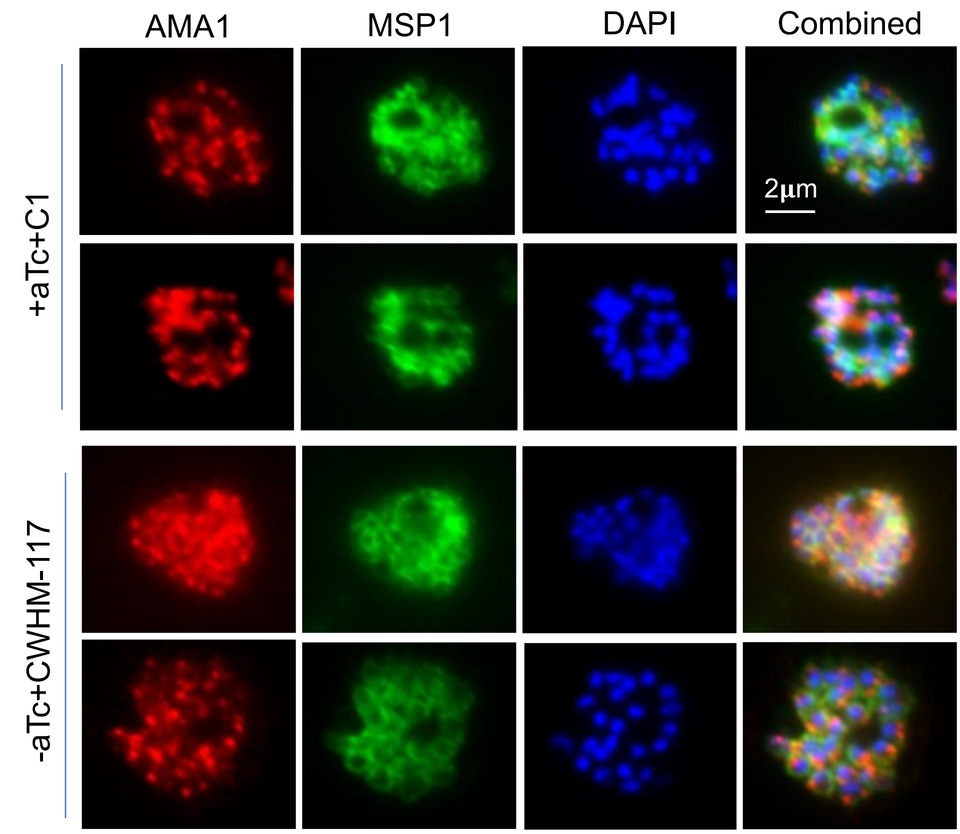

Supplementary Fig. 10. Unprocessed AMA1 accumulates in terminal organelles in *P. falciparum* schizonts. Synchronized schizonts (44 h old) from PM X^{apt} parasites were treated with C1 in presence of aTc (+aTc+C1). To knock down PM X and suppress residual activity, an aliquot of the culture was grown in absence of aTc from invasion and at 24 h post invasion, 0.5 uM CWHM-117 (-aTc+CWHM-117) was added. Samples were fixed and stained with indicated antibodies followed by IFA. Experiments were performed two times from two different biological replicates. Shown are the representative samples from each line.

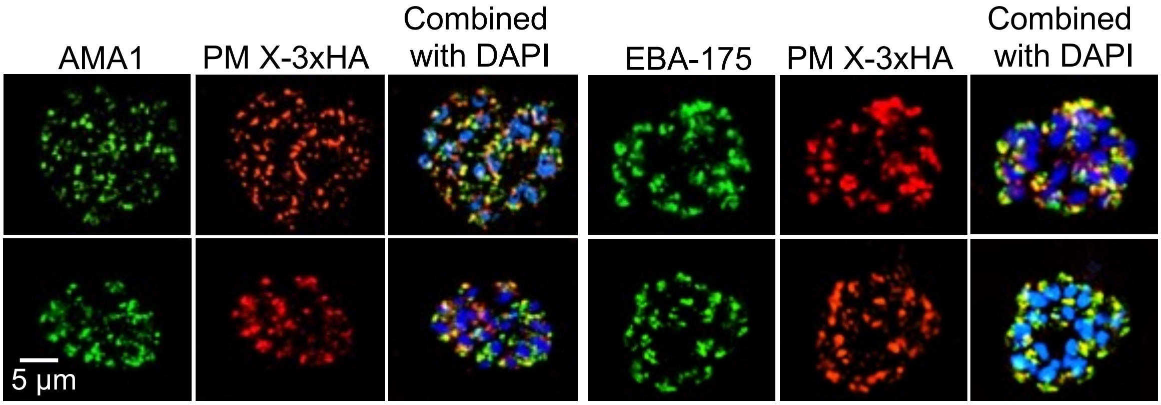

Supplementary Fig. 11. PM X partially colocalizes with the microneme markers (AMA1 and EBA175). 2-dimensional snapshots by super resolution structured illumination (SR-SIM) microscopy of parasites expressing PM X-3xHA and labeled with the indicated antibodies. Experiments were performed two times from two different biological replicates. Shown are the representative samples from each line.

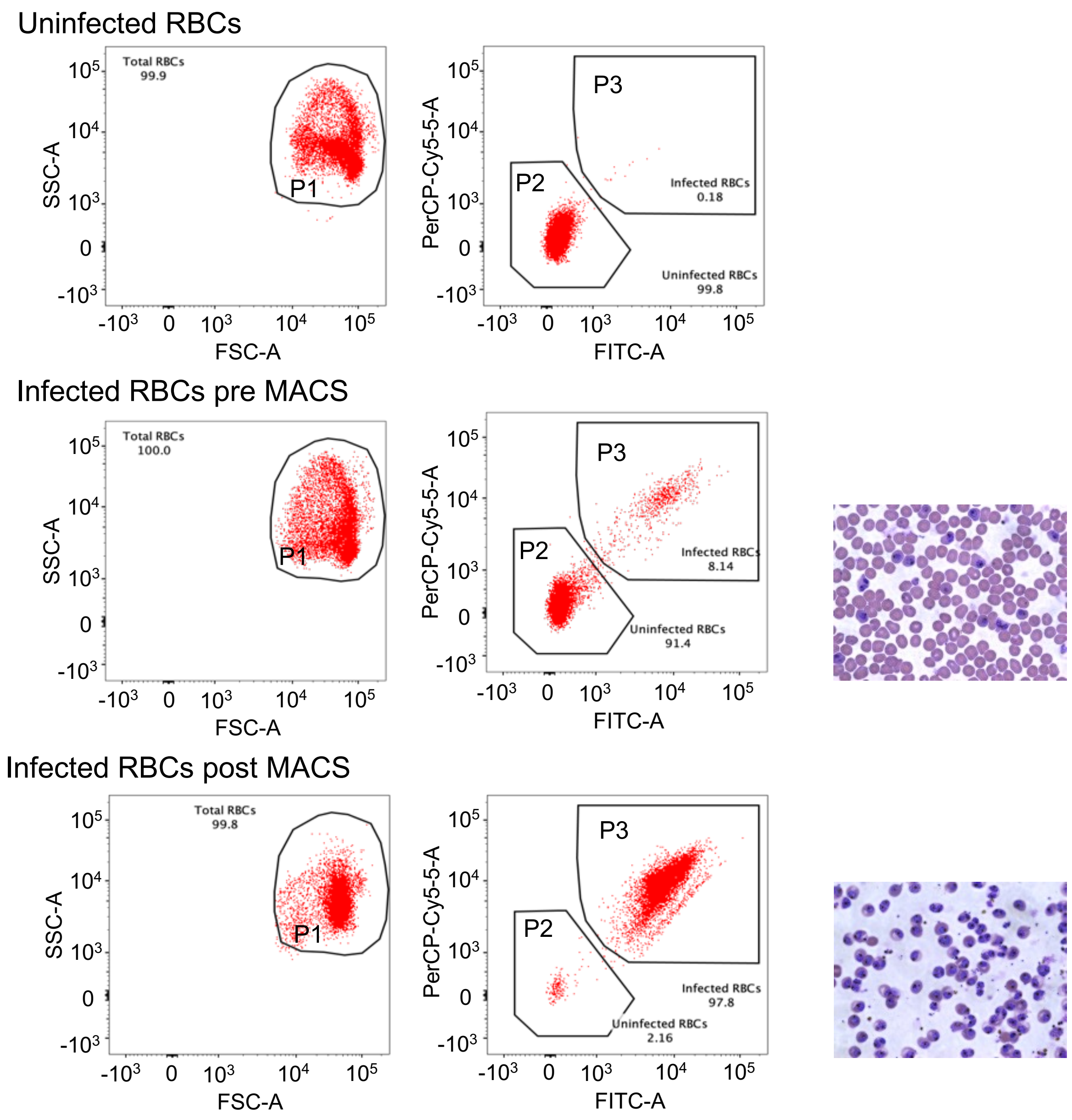

Supplementary Fig. 12. Gating strategy for determining parasitemia as depicted in fig. 2a, 5e and supplementary fig. 3. Acridine orange-stained uninfected RBCs were first gated (P1) on the basis of the characteristic forward (FSC) and side scattering (SSC) of the incident light (top, left). Total 50,000 events within P1 was then analyzed in the FITC and PerCP-Cy5 channels (top, right). The uninfected population was gated within P2 and for infected population a P3 gate was drawn. Subsequently, a sample from *P. falciparum* infected RBCs (hema stained image on right, middle panel) showed two distinct populations on a FITC vs PerCP-Cy5 dot plot. To confirm that the population in P3 represents infected RBCs, sample was magnetically purified (MACS) and analyzed by flow cytometry (lower panel). As shown in the hema stained image on bottom-right, the MACS purified sample showed 97.8% enrichment of infected RBCs. Percentage of events in P3 was counted for parasite growth assays presented in the manuscript.

Raw data for Supplementary blots/ gels

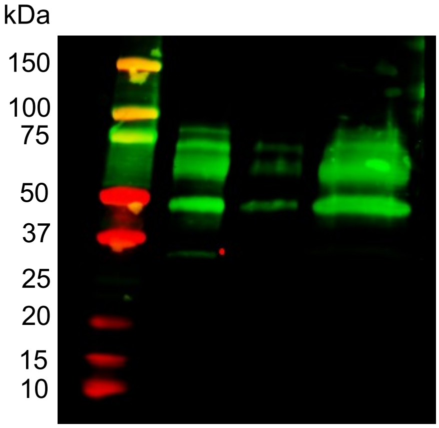

IB: anti-His

Supplementary Figure 1b

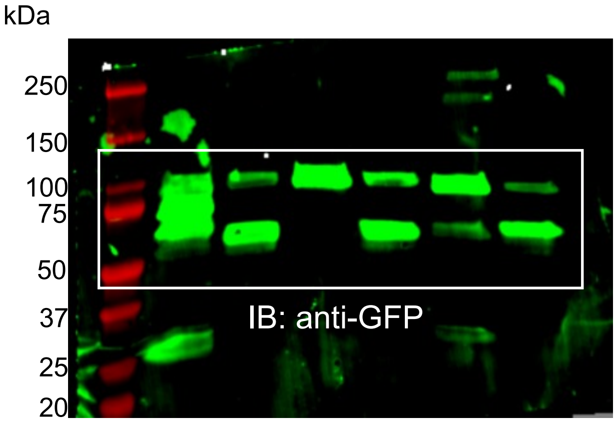

Supplementary Figure 3

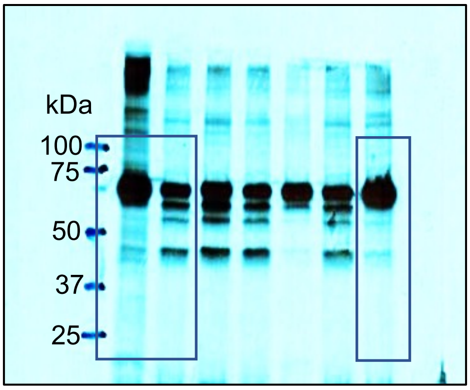

Supplementary Figure 9
