# Supplementary material for: Maturation and substrate processing topography of the Plasmodium falciparum invasion/egress protease plasmepsin X
Source: Nat Commun. 2022 Aug 4;13:4537. doi: 10.1038/s41467-022-32271-7 (PMC9352755; doi:10.1038/s41467-022-32271-7)
Supplement: Supplementary file 3 — Description of additional supplementary information files [file 41467_2022_32271_MOESM3_ESM.docx]

**Maturation and substrate processing topography of the *Plasmodium falciparum* invasion/egress protease plasmepsin X**

| Title of the file | Description |
| --- | --- |
| Supplementary data 1 | Mass spec file showing identified peptide hits from the N terminal p12 fragment (Fig. 1b, red arrow) |
| Supplementary data 2 | Mass spec file showing identified peptide hits from the C terminal p42 fragment (Fig. 1b, blue arrow) |
| Supplementary data 3 | Mass spec file showing identified peptide hits from the N terminal fragment in the NFLD mutant (Fig. 1d, grey arrow) |

Description of the Additional Supplementary Information files:
